# Modulation of Hypoxia-Inducible Factors and Vascular Endothelial Growth Factor Expressions by Superfood Camu-Camu (*Myrciaria dubia*) Treatment in ARPE-19 and Fetal Human RPE Cells

**DOI:** 10.1155/2023/6617981

**Published:** 2023-12-30

**Authors:** Ayaka Nakai, Deokho Lee, Chiho Shoda, Kazuno Negishi, Hiroyuki Nakashizuka, Satoru Yamagami, Toshihide Kurihara

**Affiliations:** ^1^Laboratory of Photobiology, Keio University School of Medicine, Tokyo, Japan; ^2^Ophthalmology, Keio University School of Medicine, Tokyo, Japan; ^3^Ophthalmology, Nihon University School of Medicine, Tokyo, Japan

## Abstract

**Background:**

Anti-vascular endothelial growth factor (anti-VEGF) therapy via intravitreal injection is an effective treatment for patients with abnormal ocular neovascularization, such as age-related macular degeneration (AMD) and diabetic macular edema (DME). However, prolonged and frequent anti-VEGF treatment is associated with a risk of local and systemic adverse events, including geographic atrophy, cerebrovascular disease, and death. Furthermore, some patients do not adequately respond to anti-VEGF therapy. Hypoxia-inducible factor (HIF) is a transcription factor that controls the expression of hypoxia-responsive genes involved in angiogenesis, inflammation, and metabolism. The HIF/VEGF pathway plays an important role in neovascularization, and the inhibition of HIF activation could be an effective biomolecular target for neovascular diseases. The demand for disease prevention or treatment using functional foods such as superfoods has increased in recent years. Few reports to date have focused on the antineovascular effects of superfoods in the retinal pigment epithelium (RPE). In light of the growing demand for functional foods, we aimed to find novel HIF inhibitors from superfoods worked in RPE cells, which could be an adjuvant for anti-VEGF therapy.

**Methods:**

Seven superfoods were examined to identify novel HIF inhibitor candidates using luciferase assay screening. We used the human RPE cell line ARPE-19 and fetal human RPE (fhRPE) to investigate the biomolecular actions of novel HIF inhibitors using quantitative PCR and western blotting.

**Results:**

Under CoCl_2_-induced pseudohypoxic condition and 1% oxygen hypoxic incubation, camu-camu (*Myrciaria dubia*) showed HIF inhibitory effects determined by luciferase assays. Camu-camu downregulated *HIF-1α* and *VEGFA* mRNA expressions in a concentration-dependent manner. Camu-camu also inhibited HIF-1*α* protein expressions, and its inhibitory effect was greater than that of vitamin C, which is present at high levels in camu-camu.

**Conclusion:**

The camu-camu extract suppressed the activation of HIF and VEGF in RPE cells. This could assist anti-VEGF therapy in patients with abnormal ocular neovascularization.

## 1. Introduction

Hypoxia induces inflammation, cell death, and abnormal angiogenesis in humans [1–3]. Hypoxia in retinal pigment epithelial (RPE) cells is one of the leading causes of age-related macular degeneration (AMD) [4–6]. Hypoxia-inducible factor (HIF) is a transcription factor for vascular endothelial growth factor (VEGF) and other hypoxia-responsive genes in RPE cells. Under normal conditions, HIF is constitutively expressed, hydroxylated by prolyl hydroxylase (PHD), and targeted by von Hippel–Lindau protein (VHL) for ubiquitination and proteasomal degradation [7]. Under hypoxia, HIF translocates into the nucleus, binds to hypoxia response elements (HRE), and induces the expression of hypoxia-responsive genes, such as VEGF, B-cell lymphoma-interacting protein 3 (BNIP3), glucose transporter 1 (GLUT1), and phosphoinositide-dependent kinase 1 (PDK1) [8, 9]. Because the VHL/HIF/VEGF pathway plays an important role in neovascularization [2, 10, 11], targeting HIFs could be an antineovascularization treatment of AMD and other retinopathies caused by angiogenesis. Hypoxia leads to HIF stabilization and hence increases levels of VEGF in RPE cells [12, 13]. In addition, HIF expression has been observed during choroidal neovascularization (CNV) in AMD patients [14–16].

We previously reported that pharmacological inhibition of HIF-1*α* or HIF-2*α* suppressed retinal neovascularization in murine models of oxygen-induced retinopathy (OIR) [17–19], known as a retinal neovascularization model, and laser-induced CNV [20–22], known as an exudative AMD model.

Humans consume a variety of nutrients in a daily life. Interests in the relationship between food and health and the prevention of diseases through food in recent years have increased as healthcare costs, life expectancy, and health concerns have been issued among people in developed countries [23, 24]. In general, superfoods are ingredients consumed for health promotion purposes for a long time, with excellent nutritional balance or exceptionally high amounts of certain nutrients [24, 25]. Medical uses of superfoods, such as the prevention of metabolic syndrome through the consumption of superfoods, have also been reported [26–28]. Previously, ten clinical trials, which investigated broccoli sprout supplementation and cardiometabolic health, showed that the dietary intake of broccoli sprouts significantly reduced systolic and diastolic blood pressures [29]. The health effect of camu-camu is still in controversial status. However, daily 70 mL of 100% camu-camu juice taken for 7 days could reduce oxidative stress markers, such as levels of urinary 8-hydroxy-deoxyguanosine and inflammatory markers, including serum levels of high-sensitivity C-reactive protein, interleukin (IL)-6, and IL-8 in male with smoking [14].

In recent fundamental studies using superfoods, the polyphenol velutin of acai fruit has been shown to downregulate HIF-1*α* expression in RAW 264.7 mouse monocyte macrophage cells [30], and wolfberry polysaccharides could inhibit HIF-1*α* expression in the mouse retina [31]. Few studies to date have focused on the antineovascular effects of superfoods on RPE cells. In this study, we screened seven superfoods (camu-camu, coconut, broccoli sprout, chia seed, hemp, maca, and cacao) that have been suggested to have efficacies in human health in interventional clinical trials [28, 29, 32–34] for new inhibitors for the HIF/VEGF pathway in RPE cells.

## 2. Materials and Methods

### 2.1. Cell Culture

The human retinal pigment epithelial cell line ARPE-19 was cultured in DMEM/F-12 (Cat #C11330500BT, Gibco, NY, USA) media with 10% FBS and 1% streptomycin-penicillin in a 5% CO_2_ atmosphere at 37°C. fhRPE cells were cultured in the cell culture medium, as previously reported [35]: MEM, *α*-modification media (Cat # M-4526, Sigma-Aldrich, MO, USA) with N1 supplement (Cat# N-6530, Sigma-Aldrich) 1 : 100 mL/mL, nonessential amino acid solution (Cat# M-7145, Sigma-Aldrich) 1 : 100 mL/mL, hydrocortisone (Cat# H-0396, Sigma-Aldrich) 20 *μ*g/L, taurine (Cat# T0625-10G, Sigma-Aldrich) 250 mg/L, and triiodo-thyronine (Cat# T-5516, Sigma-Aldrich) 0.013 *μ*g/L, 5% FBS, and 1% penicillin-streptomycin in a 5% CO_2_ atmosphere at 37°C.

### 2.2. Superfood Sample Preparation

Seven superfoods were prepared: six powdered samples of camu-camu (*Myrciaria dubia*) (Seikatsunoki, Tokyo, Japan), coconut (Arisan, Saitama, Japan), broccoli sprout (Imajin, Saitama, Japan), chia seed (Navitas organics, Novate, CA, USA), hemp (IMPLEX, Osaka, Japan), and maca (Seikatsunoki, Tokyo, Japan) dissolved in Milli-Q® water (MQ) at various concentrations. Cacao nibs were homogenized with zirconia balls (As One, Osaka, Japan) at 6,000 rpm for 20 s three times in MQ. All samples were adjusted immediately before use. Camu-camu powder used in this study was made from camu-camu pulps and contains 5,850 mg of vitamin C per 100 g.

### 2.3. Luciferase Assay Screening

ARPE-19 cells were transfected with HIF activity-dependent firefly luciferase and endogenous control CMV-Renilla luciferase using a lentivirus. We produced a stable-expression cell line “RH-ARPE19,” as previously described [17, 20]. The steps are as follows: The HIF-luciferase reporter gene (Cignal Lenti HIF-1 Reporter #336891 CLS-007L, Qiagen, Venlo, Netherlands) were transfected into ARPE-19 cells using a lentivirus. Regarding HIF-firefly luciferase, multiplicity of infection (MOI) was set to 25, and 2 × 10^4^ cells were infected with HIF-1*α*-firefly lentivirus 5 × 10^5^ TU. These cells were also cotransfected with CMV-Renilla luciferase construct as an internal control. The MOI was set to 3, and 2 × 10^4^ cells were infected with CMV-Renilla Control (Cignal Lenti CMV-Renilla Control Reporter #336891 CLS-RCL, Qiagen, Venlo, Netherlands) 6 × 10^4^ TU using a lentivirus. Infections were carried out simultaneously, and after infection, antibiotic selection was performed using puromycin and hygromycin; stable cell lines were established by cloning. Firefly/Renilla is 25/3 from the set MOI.

RH-ARPE19 cells were seeded at 1.0 × 10^4^ cells/well/70 *μ*L in white sterile HTS Transwell-96 receiver plates (Corning, NY, USA). After 24 h, the cells were treated with cobalt chloride (CoCl_2_) (200 *μ*M, cobalt (II) chloride hexahydrate; Wako, Saitama, Japan) as a proline hydroxylase (PHD) inhibitor or incubated under 1% O_2_ hypoxic conditions to stabilize HIF expression. Superfood samples (1 mg/mL) or camu-camu (1–1,000 *μ*g/mL) were added to assess the HIF inhibitory effect. After 24 h of incubation, luciferin was added to obtain a luminescence signal that reflects HIF activity. Luminescence was measured using a Dual-Luciferase® Reporter Assay System (Promega, Madison, WI, USA). In addition, 1 mM of topotecan (Cayman Chemical, Ann Arbor, MI, USA) or doxorubicin (Tokyo Chemical Industry Co., Ltd., Tokyo, Japan) was used as positive controls of HIF inhibition [36]).

### 2.4. Quantitative PCR

The effects of the camu-camu extract on the expression of HIF signaling-related genes were examined, as previously described [19, 21]. Specifically, ARPE-19 cells were seeded in 12-well plates (Corning) at 1.2 × 10^5^ cells/well/mL. After 24 h, camu-camu extract (0.1–30 *μ*g/mL) and vitamin C (1.755 *μ*g/mL, L(+)-ascorbic acid; Nacalai Tesque, Inc., Kyoto, Japan) were added to the culture medium. Thirty micrograms of camu-camu extract contains 1.755 *μ*g of vitamin C. After 7 h of incubation, the cells were dissolved in TRI reagent (MRC Global, Cincinnati, OH, USA), and RNA extraction was performed using EconoSpin columns (GeneDesign, Osaka, Japan). The columns were washed with RPE and RWT buffers (Qiagen, Hilden, Germany). cDNA was synthesized from extracted RNA using ReverTra Ace™ qPCR RT Master Mix with gDNA Remover (Toyobo Co., Ltd. Osaka, Japan). Real-time PCR was performed using THUNDERBIRD® SYBR® qPCR Mix (Toyobo Co., Ltd. Osaka, Japan) with QuantStudio 5 (Life Technologies, Carlsbad, CA, USA).

The primer sequences used were as follows: HIF-1*α* forward TTCACCTGAGCCTAATAGTCC, HIF-1*α* reverse CAAGTCTAAATCTGTGTCCTG, HIF-2*α* forward CGGAGGTGTTCTATGAGCTGG, HIF-2*α* reverse AGCTTGTGTGTTCGCAGGAA, GLUT1 forward CGGGCCAAGAGTGTGCTAAA, GLUT1 reverse TGACGATACCGGAGCCAATG, BNIP3 forward GACAGAGTAGTTCCAGAGGCAGTTC, BNIP3 reverse GTGTGCATTTCCACATCAAACAT, PDK1 forward ACAAGGAGAGCTTCGGGGTGGATC, PDK1 reverse CCACGTCGCAGTTTGGATTTATGC, VEGF forward TCTACCTCCACCATGCCAAGT, VEGF reverse GATGATTCTGCCCTCCTCCTT,* β*-actin forward GGAGGAAGAGGATGCGGCA, and *β*-actin reverse GAAGCTGTGCTATGTTGCTCTA. The fold change between the levels of different transcripts was calculated by the *∆∆*CT method.

### 2.5. Western Blotting

ARPE-19 and fhRPE cells were lysed on ice in RIPA buffer (Thermo Fisher Scientific, Waltham, MA, USA) containing a protease inhibitor cocktail (Roche Diagnostics, Basel, Switzerland) to extract the cellular proteins. Equal amounts of protein (based on the BCA assay) were treated with a sample buffer solution containing a reducing reagent (Nacalai Tesque, Inc., Kyoto, Japan). The samples were heated to 95°C for 3 min, fractionated by 10% SDS-PAGE, transferred to polyvinylidene fluoride (PVDF) membranes, and immunoblotted. Anti-*β*-actin antibody (1 : 5000, Cat #3700, Cell Signaling Technology, Danvers, MA, USA) was used as an internal standard to normalize each sample. Anti-HIF-1*α* (1 : 1000, Cat #36169, Cell Signaling Technology) and anti-HIF-2*α* (1 : 1000, Cat #NB100-122, Novus Biologicals, Centennial, CO, USA) were used as primary antibodies. The immunoblots were developed using horseradish peroxidase-conjugated secondary antibodies (1 : 5000; GE Healthcare, Princeton, NJ, USA). Signals were detected using an ECL kit (Ez WestLumi plus, ATTO, Tokyo, Japan) and imaged using chemiluminescence (ImageQuant™ LAS 4000 mini, GE Healthcare). All raw data of western blotting is available in Supplement Figure 1.

### 2.6. Statistical Analysis

Statistical significance was calculated using two-tailed Student's *t*-test for comparison of two groups. *p* values of less than 0.05 were considered statistically significant.

## 3. Results

**Figure 1:**
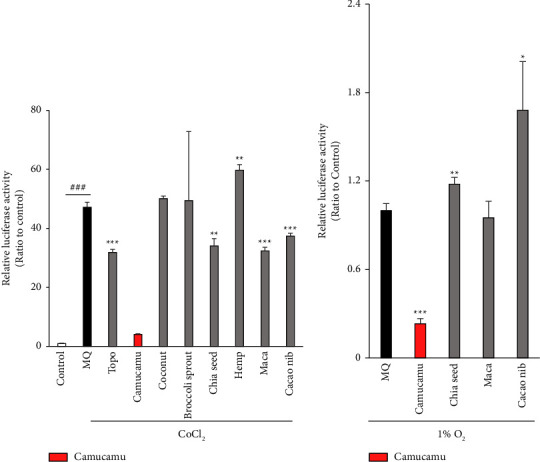
Inhibitory hypoxia-inducible factor (HIF) activity effects of superfoods. Of seven superfoods (camu-camu, coconut, broccoli sprout, chia seed, hemp, maca, and cacao nib) after the first screening, four samples (camu-camu, chia seed, maca, and cacao nib) were shown to be positive (a). After the second screening, camu-camu was identified as a new HIF inhibitor candidate (b). Quantitative analyses of the HIF-reporter luciferase assay using ARPE-19 cells (*n* = 3 per group, biological). The superfoods were added at 1 mg/mL each. (a) HIF activity induced by 200 *μ*M CoCl_2_ or (b) 1% O_2_ hypoxic incubation. The control was no induction of HIF activity. ^###^*p* < 0.001 compared with no treatment. ^*∗*^*p* < 0.05, ^*∗∗*^*p* < 0.01, and ^*∗∗∗*^*p* < 0.001 compared with MQ by (a) 200 *μ*M of CoCl_2_ treatment or (b) 1% O_2_ hypoxic incubation, respectively. The bar graphs present means with the ± standard deviation. The data were analyzed using two-tailed Student's *t*-test for comparison. MQ: Milli-Q® pure water; Topo: topotecan.

**Figure 2:**
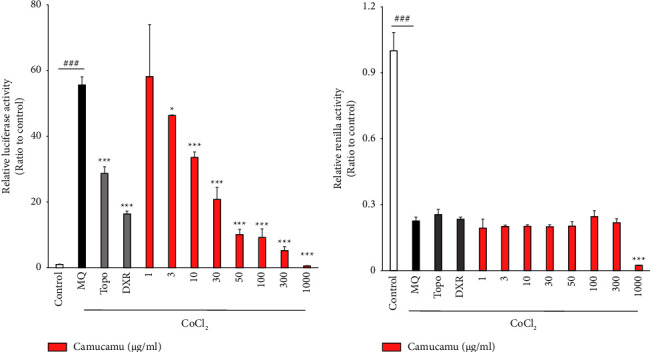
Inhibitory hypoxia-inducible factor (HIF) activity effects of camu-camu. Camu-camu was evaluated at various concentrations. (a, b) Quantitative analyses of the HIF-reporter luciferase assay using RH-ARPE19 cells (*n* = 3 per group, biological). Camu-camu was added from 1 to 1000 *μ*g/mL each. HIF activity induced by 200 *μ*M CoCl_2_. HIF activity was not induced in the control group. ^###^*p* < 0.001 compared with no treatment. ^*∗*^*p* < 0.05 and ^*∗∗∗*^*p* < 0.001 compared with MQ by 200 *μ*M of CoCl_2_ treatment. (a) Relative luciferase activity reflecting HIF-1*α* activity. (b) Relative Renilla activity reflecting cell viability. The bar graphs present means with  ± standard deviation. The data were analyzed using two-tailed Student's *t*-test for comparison. MQ: Milli-Q® water; Topo: topotecan; DXR: doxorubicin.

RH-ARPE19 cells were treated with seven superfood samples to test for the HIF inhibitory activity. For the first luciferase assay screening, 200 *μ*M CoCl_2_ was used to induce the HIF activity, and 1 mM of topotecan was used as the positive control for HIF inhibition [37, 38] (Figure 1(a)). Compared to the control containing only pure water, the relative luciferase activity was increased in the MQ group loaded with CoCl_2_-induced pseudohypoxic conditions (Figure 1(a)). The four superfood samples camu-camu, chia seeds, maca, and cacao nibs exhibited HIF-suppressive effects compared to MQ (Figure 1(a)) under CoCl_2_-induced pseudohypoxic conditions. In the second luciferase screening, to better mimic hypoxic conditions, we used 1% O_2_ hypoxic incubation to stabilize HIF expression (Figure 1(b)). The four superfood samples selected by the first trial of the luciferase assay screening were used in the second luciferase screening (Figure 1(b)). Camu-camu had a statistically significant inhibitory effect on HIF activation. Camu-camu treatment inhibited the HIF activity under CoCl_2_-induced pseudohypoxic conditions in a dose-dependent manner (from 1 to 1,000 *μ*g/mL) (Figure 2(a)). Cell toxicity of camu-camu treatment was increased between 300 and 1000 *μ*g/ml regarding internal control Renilla expression (Figure 2(b)). From the series of screening tests, we found that camu-camu may have the potential to destabilize HIF expression in human RPE cells.

**Figure 3:**
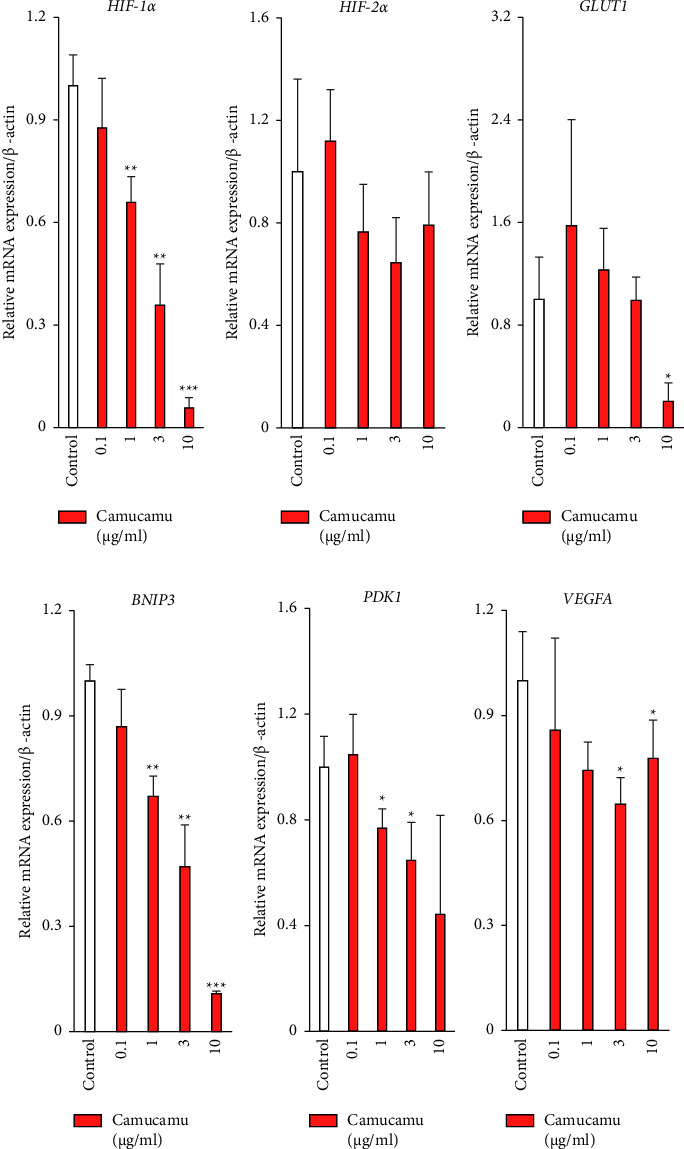
Suppression of hypoxia-responsive gene expressions by camu-camu. (a–f) Quantitative analyses (*n* = 3–5 per group, biological) show significant changes in *HIF-1α*, *GLUT1, BNIP3*, *PDK1*, and *VEGFA* mRNA expression after 7 hours under camu-camu treatment from 0.1 *μ*g/mL to 10 *μ*g/mL each in ARPE-19 cells. ^*∗*^*p* < 0.05, ^*∗∗*^*p* < 0.01, and ^*∗∗∗*^*p* < 0.001 compared with no treatment. The bar graphs present means with ± standard deviation. The data were analyzed using two-tailed Student's *t*-test for comparison.

We then investigated the effect of camu-camu treatment on *HIF-1α*, *HIF-2α*, and HIF downstream-target hypoxia-responsive gene expressions [39, 40] in ARPE-19 cells (Figure 3). After 7 hours of camu-camu treatment, the expression levels of *HIF-1α*, *GLUT1, BNIP3*, *PDK1*, and *VEGFA* were significantly downregulated under normal culture conditions (Figures 3(a), 3(d)–3(f)).

**Figure 4:**
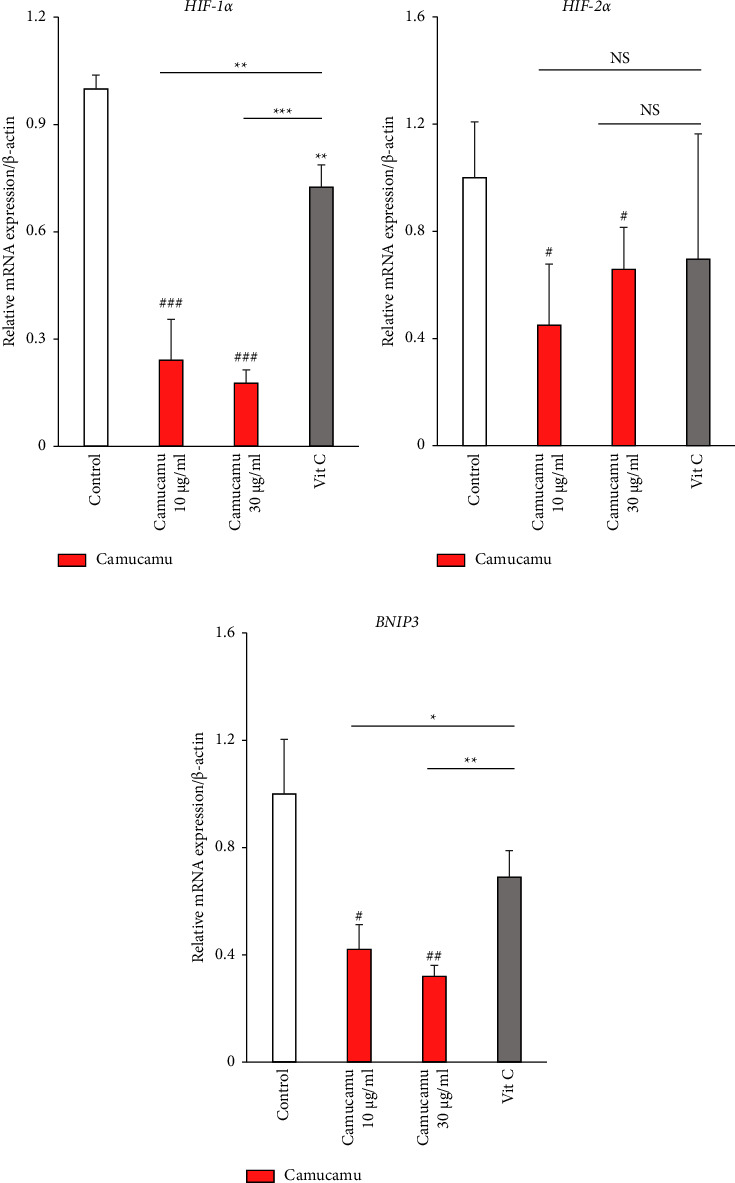
Comparison of hypoxia-responsive gene expression by camu-camu and vitamin C. Quantitative analyses (*n* = 3–5 per group, biological) using ARPE-19 cells reveal significant changes in *HIF-1α*, *HIF-2α*, and *BNIP3* mRNA expression after 7 hours of 10 *μ*g/mL and 30 *μ*g/mL camu-camu treatment. Vitamin C was added at 1.755 *μ*g/mL. Low-dose camu-camu treatment comprised 10 *μ*g/mL of camu-camu, and high-dose camu-camu treatment (30 *μ*g/mL) contained 1.755 *μ*g/mL of vitamin C. *HIF-1α*, *HIF-2α*, and *BNIP3* mRNA-suppressive effects of camu-camu treatment were generally greater than those of vitamin C treatment. ^#^*p* < 0.05, ^##^*p* < 0.01, and ^###^*p* < 0.001 compared with no treatment. ^*∗*^*p* < 0.05, ^*∗∗*^*p* < 0.01, and ^*∗∗∗*^*p* < 0.001 compared with camu-camu treatments and vitamin C treatment. NS: no significant. The bar graphs present means with ± standard deviation. The data were analyzed using two-tailed Student's *t*-test for comparison.

**Figure 5:**
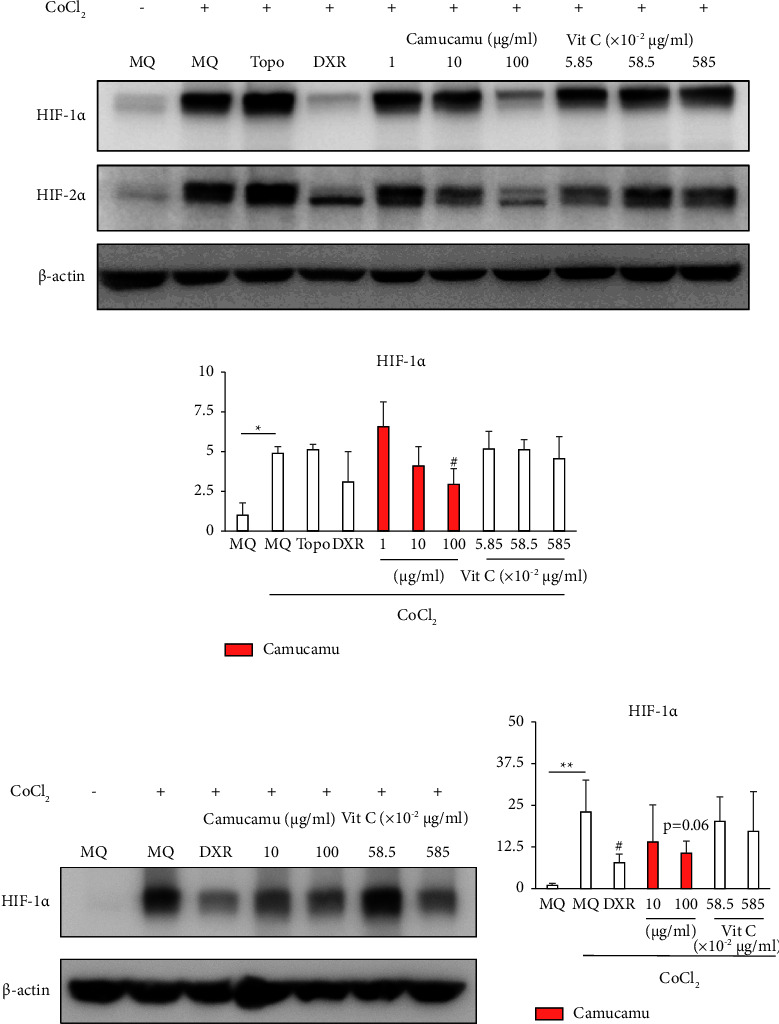
Comparison of camu-camu and vitamin C effects on suppression of HIF-1*α* protein expression. HIF-1*α* activity induced by 200 *μ*M CoCl_2_ and suppression by doxorubicin. Treatment with 1 *μ*g/mL camu-camu comprises 5.85 ×10^−2^ *μ*g/mL of vitamin C. (a) Camu-camu suppresses HIF-1*α* protein expression in ARPE-19 cells. (b) Quantification of the blots shows that the administration of camu-camu suppressed increased HIF-1*α* protein expression under CoCl_2_ in ARPE-19 cells (*n* = 3, biological and technical). (c) HIF-1*α* expression under CoCl_2_ in fhRPE cells. (d) Quantification of the blots in fhRPE (*n* = 3, biological and technical). ^#^*p* < 0.05 compared with CoCl_2_ + MQ. ^*∗*^*p* < 0.05 and ^*∗∗*^*p* < 0.01 compared with no CoCl_2_ treatment. The bar graphs present means with ± standard deviation. The data were analyzed using two-tailed Student's *t*-test for comparison. MQ: Milli-Q® pure water; Topo: topotecan; DXR: doxorubicin; Vit C: vitamin C.

Camu-camu is a vitamin C-rich fruit, and the red-stage fresh matter contains 1.88%–2.06% vitamin C by weight [41, 42]. The antioxidant properties of vitamin C are well known [43], and it can also reduce HIF-1*α* expression in vivo [44, 45]. Therefore, we compared the HIF-1*α* and HIF-2*α* inhibitory effects of camu-camu with those of vitamin C. *HIF-1α* and *HIF-2α* mRNA expressions were significantly reduced by high- and low-dose camu-camu treatments (Figures 4(a) and 4(b)). The effects of high- and low-dose camu-camu treatment on *HIF-1α* and *BNIP3* mRNA expressions were greater than those of vitamin C treatment (Figures 4(a) and 4(c)). As high-dose camu-camu treatment (30 *μ*g/ml) contains 1.755 *μ*g/ml of vitamin C, we selected 1.755 *μ*g/ml of vitamin C for vitamin C treatment (Figure 4). We examined the effects of the camu-camu extract on the expression of HIF-1*α* and HIF-2*α*. Camu-camu noticeably inhibited CoCl_2_-dependent induction of HIF-1*α* in a dose-dependent manner in ARPE-19 cells (Figures 5(a) and 5(b)). Camu-camu also had an HIF-1*α* inhibiting tendency in fhRPE cells (Figures 5(c) and 5(d)). The suppressive effects of camu-camu treatment on HIF protein expression were stronger than those of vitamin C treatment (Figure 5(b)).

## 4. Discussion

In this study, we found that camu-camu treatment could inhibit the stabilization of nonphysiologic HIF-1*α* proteins in ARPE-19 cells. Among the seven superfood candidates, camu-camu was found to be a novel HIF inhibitor based on luciferase assay screening under pseudohypoxic conditions using CoCl_2_ and 1% O_2_ (Figure 1). Camu-camu is a native Amazonian bush-bearing, round, redberry-like fruit. Camu-camu pulps are used not only in the Amazon region but also in Japan and Europe as juice, sherbet, and extracts [41]. Camu-camu contains natural antioxidants, such as vitamin C, carotene, phenolic compounds, flavonols, anthocyanins, ellagic acid conjugates, ellagitannins, gallic acid derivatives, and proanthocyanidins [41, 42, 46]. These bioactive substances have been reported to possess antioxidant and free radical scavenging abilities [47–49]. Camu-camu has been reported to contain approximately 1,882–2,061 mg of vitamin C in 100 g of fresh mature fruit [46, 50]. The camu-camu powder used in this study contained 5,850 mg of vitamin C per 100 g, indicating that 5.85% of the powder contained vitamin C by weight.

Vitamin C is a strong antioxidant [43] that inhibits HIF expression in cancer [51] and lens epithelial cells [52]. Furthermore, the mode of action of vitamin C on HIF-1*α* suppression has been suggested to involve in prolyl hydroxylation [45]. The camu-camu extract suppressed HIF-1*α* and HIF-2*α* expressions to a greater extent than vitamin C in ARPE-19 cells (Figures 4 and 5). These results suggest that components other than vitamin C in camu-camu may synergize with the inhibitory effects of HIF. In our current study, water-soluble substances in camu-camu were only focused. Therefore, it might be required to investigate HIF-inhibitory effects of hydrophobic compounds in camu-camu (such as ellagic acid conjugates and gallic acid derivatives). The ellagic acid has been reported to have HIF-1*α* suppressive effects on the human urinary bladder carcinoma cell line (ECV304) [53]. Aqueous HIF-inhibitory substances can reach the choroidal blood vessels that nourish RPE cells, as abundant blood flows into the choroid blood vessels from the short posterior ciliary artery, the long posterior ciliary artery, and the anterior ciliary artery which are from the internal carotid artery. Taken together, the investigation on substances in camu-camu that may have additional HIF-inhibitory effects will be further studied.

Intravitreal anti-VEGF therapy is an important treatment option for patients with vision loss due to abnormal neovascularization, including AMD, macular edema secondary to retinal vein occlusion (RVO), diabetic macular edema (DME), myopic choroidal neovascularization (mCNV), and retinopathy of prematurity (ROP). More than 2.5 million intravitreal injections are used annually in the United States [54]. Anti-VEGF therapy appears to be an effective treatment for the retina; however, prolonged and/or frequent treatments may be associated with an increased risk of ocular local and/or systemic adverse events, including geographic atrophy [55], cerebrovascular disease, and death [56]. Occasionally, patients do not respond adequately to anti-VEGF therapy [57, 58]. Therefore, it is necessary to explore treatment options other than anti-VEGF therapy for these diseases. In this regard, our camu-camu extract might be helpful.

Increased HIF-1*α* expression in RPE cells promotes the production of VEGF, and increased VEGF expression promotes the development of abnormal neovascularization [59–61]. Muller cells have possibilities to play an important role in the production of VEGF and HIF-1*α*, which are associated with inflammation of the inner retinal layers, such as in diabetic retinopathy [62]. Although we focused on HIF and VEGF expressions in RPE cells in our current study, it may be necessary to consider Muller cells for the further work. Based on the role of HIF-1*α* in angiogenesis, HIF-1*α* may represent a molecular therapeutic target for ocular neovascularization diseases in addition to VEGF, as noted in previous reports [63–65]. We showed that the camu-camu extract inhibited HIF-1*α* and *HIF-2α* expressions in ARPE-19 cells. The *HIF-2α* mRNA level was decreased by the 10 *μ*g/ml and 30 *μ*g/ml camu-camu treatments (Figure 4). The 10 *μ*g/ml and 30 *μ*g/ml camu-camu treatments inhibited the transcription of *HIF-2a*, but the translation is unknown from this experiment because we were unable to detect translation changes in HIF-2*α* protein. Regarding HIF-1*α*, the camu-camu treatments inhibit transcription and translation (Figures 3–5). VEGF and its receptors VEGF receptor-1 and VEGF receptor-2 are directly induced by HIF-2*α* under hypoxic conditions through their identified HRE [66, 67]. Inhibition of excessive *HIF-2α* expression may also be protective against ocular neovascularization and RPE atrophy since a relationship between HIF-2*α* and angiogenic retinopathy has been suggested [16]. Particularly, in retinal disease characterized by neovascularization as a result of severe tissue hypoxia, such as AMD [16] or proliferative diabetic retinopathy (PDR) [68], HIF-2*α* is expressed in the subretina in patients. Deferoxamine (DFO), an iron chelator, causes RPE atrophy as adverse effects. Clinically inhibiting upregulation of HIF-2*α* by *α*-ketoglutarate relieved DFO-related RPE atrophy [69]. Camu-camu, which suppresses both HIF-1*α* and HIF-2*α* expressions in RPE cells, could become an adjuvant therapy to assist current treatments for patients with abnormal neovascularization and subsequent RPE atrophy. Because camu-camu can readily be consumed in the form of juice or food, it is considered acceptable to patients mentally and economically. However, the dosage and administration need to undergo further study.

## 5. Conclusions

Although the in vivo effects need to be further investigated, we found that among the seven superfood candidates, camu-camu treatment inhibited upregulation of HIF/VEGF expressions in ARPE-19 cells. Camu-camu could become an adjuvant therapy to assist anti-VEGF therapy in patients with abnormal neovascularization and subsequent RPE atrophy in an era of rising expectations regarding functional foods and superfoods.

## Data Availability

The data used to support the findings of this study are available from the corresponding author upon request.

## Supplementary Materials

Supplementary Materials Supplement Figure 1: Western blotting raw data. HIF-1*α*, HIF-2*α* and *β*-actin protein expression in (A–C) ARPE-19 cells and (D–F) fhRPE cells. The experiments were repeated individually, and quantified using Image J (Open-source tools: https://imagej.nih.gov/ij/download.html). The Figure 5B was created from the S1 (A–C) and the Figure 5D was from S1 (D–F).
