# Supplementary material for: Modulation of Hypoxia-Inducible Factors and Vascular Endothelial Growth Factor Expressions by Superfood Camu-Camu (Myrciaria dubia) Treatment in ARPE-19 and Fetal Human RPE Cells
Source: J Ophthalmol. 2023 Dec 30;2023:6617981. doi: 10.1155/2023/6617981 (PMC10771337; doi:10.1155/2023/6617981)
Supplement: Supplementary Materials — Supplement Figure 1: Western blotting raw data. HIF-1α, HIF-2α and β-actin protein expression in (A–C) ARPE-19 cells and (D–F) fhRPE cells. The experiments were repeated individually, and quantified using Image J (Open-source tools: https://imagej.nih.gov/ij/download.html). The Figure 5B was created from the S1 (A–C) and the Figure 5D was from S1 (D–F). [file 6617981.f1.zip › Supplement Figure 1 Figure legends HIF inhibitor camucamu extract in RPE 20231216.docx]

**Supplement Figure 1. Western blotting raw data.** HIF-1α, HIF-2α and β-actin protein expression in (A-C) ARPE-19 cells and (D-F) fhRPE cells. The experiments were repeated individually, and quantified using Image J (Open-source tools: https://imagej.nih.gov/ij/download.html). The Figure 5B was created from the S1(A-C) and the Figure 5D was from S1(D-F).
